# A Case of Thiazide-induced Hypokalemic Paralysis

**DOI:** 10.5811/cpcem.2019.3.42062

**Published:** 2019-05-20

**Authors:** Elizabeth Schell, Joshua Pathman, Richard Pescatore, Pollianne W. Bianchi

**Affiliations:** * Drexel University College of Medicine, Philadelphia, Pennsylvania; † Crozer-Keystone Health System, Department of Emergency Medicine, Upland, Pennsylvania

## Abstract

We describe the case of a patient presenting with odd neurologic symptoms initially thought to represent somatization who was found to have critical hypokalemia manifesting as hypokalemic non-periodic paralysis. It was determined that the patient had baseline hypokalemia as a function of alcohol abuse, exacerbated by self overmedication with hydrochlorothiazide for elevated blood pressure readings at home. The diagnosis was suspected when an electrocardiogram was obtained demonstrating a pseudo-prolonged QT interval with ST depression, consistent with T-U wave fusion and a QU interval with an absent T wave.^1^ The patient received oral and intravenous potassium and magnesium supplementation with resolution of symptoms.

## INTRODUCTION

We report a rare and unusual case of hypokalemic non-periodic paralysis (HNPP) precipitated by thiazide diuretic overuse in the setting of alcoholic malnutrition. HNPP associated with thiazide diuretics is extremely rare and reported only sparingly in the literature. Unique features of this case include the patient’s non-specific presentation, pathognomonic electrocardiogram (ECG) changes, and the severity of the patient’s hypokalemia and associated hypomagnesemia. The patient had complete resolution of symptoms with electrolyte repletion in the emergency department (ED). We discuss the incidence and mechanism of hypokalemic paralysis and a detailed description of the patient’s presentation as a reminder for emergency physicians to consider HNPP in the differential diagnosis of patients treated with thiazide diuretics presenting with neurologic symptoms.

## CASE REPORT

A 53-year old female with a history of alcohol abuse, anxiety, and hypertension presented to the ED with a chief complaint of “I think I’m having a stroke.” The patient reported inability to move her face or hands since awaking that morning, about 30 minutes prior to arrival. She was immediately assessed by physician and nursing staff after triage personnel initiated a rapid stroke evaluation.

On physical exam, the patient held her mouth immobile and partly open, even while talking. On neurologic exam, the patient’s hands and wrists appeared to be flaccid, with wrists held in passive flexion. Passive range of motion of all extremities was normal with no spasticity. Muscle strength was assessed at 1/5 in wrist flexion and extension, and 4/5 throughout the remainder of the bilateral extremities. She was unable to comply with cerebellar testing due to weakness. There was no clonus. The rest of the physical exam was unremarkable, including vital signs, which demonstrated a heart rate of 96 beats per minute, blood pressure of 136/88 millimeters of mercury, respirations of 16 breaths per minute, and pulse oximetry of 99% on room air. The patient was afebrile with an oral temperature of 97.6 degrees Fahrenheit.

On further history, the patient reported daily moderate alcohol abuse of 2–3 glasses of wine, with her last drink the evening prior. She had been poorly compliant with outpatient primary care follow-up and had not seen her primary care physician (PCP) in nine months. However, she had received multiple refills of her prescribed hydrochlorothiazide (HCTZ) 25 milligrams (mg) twice daily, as well as sertraline 100mg daily, her only medications. The patient admitted to intermittent medication compliance, but stated that she had been taking extra doses of HCTZ recently due to elevated blood pressures noted on home sphygmomanometry, with an estimated daily dose of 50–75 mg of HCTZ.

The differential diagnosis was considered to be broad, including an anxiety reaction, somatization, intoxication, extrapyramidal side effects due to sertraline, or other metabolic derangements. Given her neurologic examination, a cerebral vascular accident was thought to be unlikely. Believing anxiety, somatization, and extrapyramidal side effects to be more likely, the treating physician administered 25mg of intramuscular diphenhydramine to the patient without any significant improvement. Laboratory studies were drawn and sent and an ECG obtained (Image).

The patient’s ECG was thought to be concerning for hypokalemia, which was confirmed when serum chemistry revealed a potassium level of 1.8 milliequivalents per liter (mEq/L) (normal 3.5–5.0 mEq/L). Additionally, the patient’s magnesium level was found to be critically low at 0.8 mEq/L (normal 1.5–2.5 mEq/L). Serum calcium level was 8.1 mg/deciliter (dL) with an albumin level of 2.9 grams (g)/dL (normal 3.4 – 5.4 g/dL). Corrected serum calcium was calculated to be 9.0 mg/dL (normal 8.5 – 10.2 mg/dL). Serum ethanol was undetectable and urine drug screen was negative. The patient’s sodium level was 134 mEq/L (normal 135–145 mEq/L). Her complete blood count, thyroid stimulating hormone, and creatine kinase were within normal limits. She had normal renal function. A presumptive diagnosis of HNPP was made and potassium and magnesium correction started through oral and intravenous (IV) routes.

Following administration of 40 mEq of oral potassium chloride, 20 mEq of IV potassium chloride, 800 mg of oral magnesium oxide, and 2 grams of IV magnesium sulfate, the patient had complete resolution of her symptoms and narrowing of corrected QT interval. Potassium and magnesium supplementation was continued and the patient was admitted for evaluation by internal medicine and nephrology.

The patient received additional magnesium and potassium supplementation, and subsequent serum chemistries demonstrated resolution of hypokalemia and hypomagnesemia. She was evaluated by nephrology, who felt that the cause of the patient’s electrolyte abnormalities was likely multifactorial, a function of diuretic abuse and alcoholic malnutrition. While congenital channelopathies were considered and thought to be possible, genetic testing was deferred. The patient’s HCTZ was discontinued in lieu of amlodipine 10mg daily and the patient started on 20 mEq daily potassium chloride supplementation. She was discharged to home and advised to obtain repeat laboratory testing with her PCP.

## DISCUSSION

Hypertension is a major cause of morbidity and mortality in the United States. Thiazide diuretics are the most commonly prescribed anti-hypertensive medication. Thiazides are typically very safe and effectively lower blood pressure with few side effects or adverse events.^2^ Potential side effects do exist, however, including renal potassium wasting leading to hypokalemia. Thiazides work via disruption of sodium/chloride co-transporters in the distal tubule leading to increased sodium infiltrate. This increase leads to a surge in sodium resorption distally via sodium-potassium/hydrogen counter transporters and a subsequent urinary loss of potassium. This loss of potassium and hydrogen ions can potentially lead to hypokalemia and metabolic alkalosis, a common co-occurrence.^3^

**CPC-EM Capsule**

What do we already know about this clinical entity?

Hypokalemic paralysis is associated with genetic channelopathy or thyrotoxicosis. Symptoms of weakness or paralysis lead to respiratory failure and cardiac arrhythmias.

What makes this presentation of disease reportable?

We present an unusual case of hypokalemic non-periodic paralysis caused by potassium depletion that was related to drug side effects and compounded by malnutrition.

What is the major learning point?

An electrocardiogram may give insight into the cause of vague weakness of unclear etiology, such as that caused by otherwise-benign thiazide diuretics.

How might this improve emergency medicine practice?

This case reinforces the need for a high index of suspicion for electrolyte abnormalities in patients with alcohol use disorder or who present with vague weakness.

Given their mechanism of action, it is well-documented that non-potassium sparing diuretics, including thiazides, can cause hypokalemia. The first case of hypokalemic paralysis caused by thiazides was documented in 1958 in a man instructed to take 250 mg HCTZ twice daily for hypertension.^4^ Potassium wasting has since been found to be dose-dependent, and current guidelines do not recommend a patient take more than a total of 100 mg HCTZ per day.

Even with reduction in dosing, hypokalemia is a potential side effect in certain patients taking thiazides, and it is associated with increased mortality in hospitalized patients. Mild hypokalemia (between 3.5 and 3.0 mEq/L) is typically asymptomatic. Below 3.0 mEq/L, patients may experience muscle cramping, muscle weakness, myalgias, and malaise. In severe hypokalemia (less than 2.5 mEq/L), symptoms can include ECG changes, arrhythmias, and paralysis. 5 If left untreated, severe hypokalemia can lead to respiratory failure and fatal dysrhythmias. However, even in severe cases, up to 50% of patients are asymptomatic.^6^

In the presented case, the patient presented with paralysis and ECG changes due to hypokalemia from potassium wasting due to medication-induced renal wasting in the presumed setting of alcoholic malnutrition. Hypokalemia as well as hypomagnesemia are frequently present in the alcoholic patient with prevalence of 12% and 30%, respectively.^7^ Hypokalemic paralysis caused by loss of total body potassium is typically referred to as hypokalemic non-periodic paralysis. Another type of hypokalemic paralysis, caused by inherited channelopathies, is called hypokalemic periodic paralysis. In contrast to potassium wasting, the channelopathy causes a rapid shift of potassium into cells and subsequent paralysis.^8^ Genetic channelopathy testing was considered in our patient, but was ultimately declined because of the other likely causes of hypokalemia including diuretic use and alcoholism.

As stated above, the overwhelming majority of patients taking thiazides do not develop clinically significant hypokalemia. It is not completely understood why some patients are more sensitive to the potassium lowering effects of thiazides, but gastrointestinal absorption likely plays a role. Differences in dietary potassium intake and other factors affecting potassium absorption, such as chronic alcohol use, laxative use, and bulimia nervosa put a patient at higher risk for developing hypokalemia.^9^

It was the ECG obtained in this case that sparked initial concern for hypokalemia in this patient. Several classic hypokalemia ECG findings are noted in this case including diffuse ST depressions, as well as diffuse flattening of the T waves with the beginnings of T wave inversions in some lateral leads. Prominent U waves are seen following the flattened T waves in the precordial and lateral leads. The flattening of the T wave in conjunction with the prominent U wave can give the impression of an isolated prolonged QT, which should not be confused with the ECG manifestations of hypocalcemia. Not well visualized in this case is the increase in length and amplitude of the P wave or the significant T wave inversions which give a biphasic appearance to the TU segment.

As outlined previously, thiazide-induced HNPP is driven primarily by whole body depletion of potassium. Successful treatment in this case (and severe hypokalemia in general) focuses on rapid repletion of potassium in addition to magnesium. Oral formulations of potassium chloride are generally preferred to their IV counterparts; however, the IV route should be used in patients unable to tolerate oral medication or with cardiac arrhythmias or acute respiratory failure.^10^ This patient received rapid IV infusion due to ECG changes concerning for a developing cardiac arrhythmia.

Equally important to potassium administration is the requirement for magnesium coadministration to correct the hypomagnesemia that frequently accompanies hypokalemia. Potassium chloride administration is futile in the untreated hypomagnesemic patient as magnesium is critical in maintaining intracellular potassium levels.^11^ Recommendations on repletion vary; however, administration of 2g IV magnesium sulfate, as was done in this case, is an accepted initial regimen. Further repletion will often be required over the following 24 hours.^12^

## CONCLUSION

While hypokalemic periodic paralysis associated with an inherited genetic mutation or thyrotoxicosis is an infrequent but well-documented entity, hypokalemic paralysis associated with thiazide use – termed hypokalemic non-periodic paralysis – has little discussion in the literature. This case report demonstrates how a patient presentation that could have been dismissed as somatization was actually caused by a severe electrolyte abnormality, which can be associated with cardiac arrhythmia and respiratory failure. Hypokalemic non-periodic paralysis should be considered in the differential diagnosis for patients presenting with paralysis who are prescribed thiazide diuretics or other potassium-lowering medications. Clinicians should have a low threshold to investigate for hypokalemia in patients presenting with symptoms suggestive of paralysis.

## Figures and Tables

**Image:**
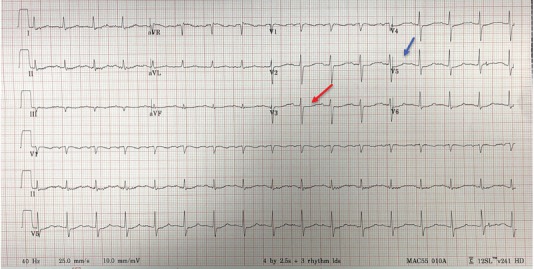
Electrocardiogram demonstrating a pseudo-prolonged corrected QT interval (522 milliseconds) with ST depression (red arrow), consistent with T-U wave fusion and a QU interval with an absent T wave (blue arrow).
